# The mental health of rural older adults: the role of the built environment in Jintang County

**DOI:** 10.3389/fpubh.2023.1203675

**Published:** 2023-06-23

**Authors:** Ping Liang, Yan Wang, Tong Wang

**Affiliations:** ^1^Humanities and Law School, Chengdu University of Technology, Chengdu, China; ^2^Department of Engineering Management, Sichuan College of Architectural Technology, Deyang, China; ^3^Faculty of Architecture and Built Environment, Delft University of Technology, Delft, Netherlands

**Keywords:** mental health, older adults, binary logistic model, rural areas, built

## Abstract

The mental health of older adults has become one of the major health challenges facing society today, which has attracted wide concerns from scholars in urban areas, but research in rural areas has been neglected. Therefore, this paper took rural older adult residents of 11 sample villages in Jintang County, Chengdu City, Sichuan Province, as the research object. After controlling the demographic characteristics of older adults in rural areas, this paper attempted to explore the effects of the rural built Environment on the mental health of older adults. Through field investigation in the sample villages, 515 valid questionnaires were obtained. The results from the Binary Logistic Regression Model show that good marital status, physical health, education level, well-constructed roads, and safe neighborhoods had significant positive effects on the mental health of rural older adults. Rural older adults who prefer to walk, cycle, and use public transport have better mental health, and accessibility to the periodic market, health station, bus station, village committee, supermarket, and the main road is positively correlated with the mental health of rural older adults, while the distance from home to the town center and coach terminal has a significant negative impact on the mental health of rural older adults. The research results provide a theoretical reference for further construction of rural aging environments.

## 1. Introduction

**Figure 1:**
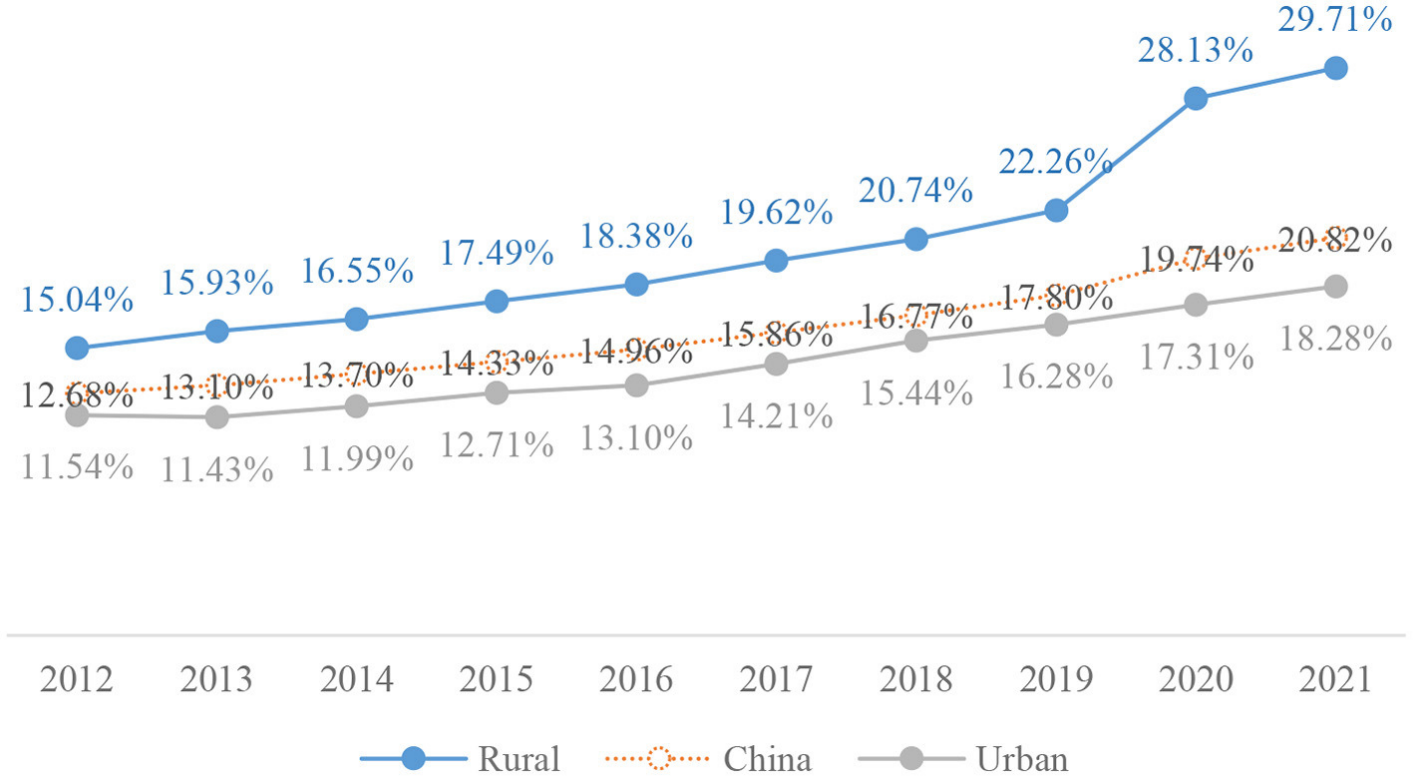
Data on China's older adult population.

According to China's sixth and seventh population censuses, the older adult population was 178 million and 264 million, respectively. The proportion of the older adult population was 13.26% and 18.70%, respectively. The proportion of older adults increased by 5.44% (1, 2). With the increasing severity of aging, “older adults' health” has become an important issue in social development. Compared with young people, older adults may be more influenced by the surrounding environment due to low mobility (3). Due to their low socioeconomic status and degraded physical functioning, older adults are more likely to suffer from mental health problems (4, 5). Therefore, research on “aging health” has become an important part of the national strategy (6). The problem of aging and aging health is more prominent in rural China than in urban regions. According to China's sixth and seventh censuses, the older adult population was 0.99 million and 1.21 million, respectively, in rural China, which accounts for 14.98% and 23.81%, respectively. Compared to the census in 2010, the proportion of people aged 60 and above in rural areas rose by 8.83 percentage points (China Statistical Yearbook in 2010 and 2021) in China's seventh census (more data on China's older adult population is shown in Figure 1). The rural aging level is significantly higher than the national aging level. Due to the relatively backward economic development in rural areas, there is a huge gap between rural and urban areas in medical resources. Meanwhile, with the urbanization of China, the rural hollowing is becoming more and more serious. Rural labor forces go out for work and the proportion of permanent rural older adult population is actually higher. More attention should be paid to the health of older adults left behind in rural areas.

In 2003, the American Journal of Public Health published a collection whose theme was “The Built Environment and Health.” In the same year, the American Journal of Health Promotion also published a collection which was titled “Health Promotion and Community Design.” As the two authoritative journals in the field of health research, the two special issues have greatly impacted the field of health research, indicating that the impact of the built environment on health has been recognized by the mainstream academic world (7). In recent years, the relationship between the built environment and mental health has also been extensively studied in the general population (8, 9). However, they mainly focus on urban areas, and there are few relevant studies on rural areas, especially on the rural older adult population. Therefore, this study focuses on the mental health problems of older adults in rural areas, attempts to explore the role of the rural built environment, and provides a theoretical reference for the construction of an age-appropriate rural environment in the process of China's new rural construction.

The rest of this paper is structured as follows: the second section reviews the current research in this field. Data collection and variable description are presented in Section 3. The fourth section discusses the research results. Finally, the fifth part summarizes this research.

## 2. Literature review

### 2.1. Built environment and the mental health of older adults

In recent years, the effect of the built environment on residents' mental health mainly focused on two aspects: objective and subjective built environment (10). Existing research shows that the relationship between the built environment and mental health is widely confirmed in the general population (9). Yang et al. (11) believe that the accessibility to green space in parks is the most important factor that affects the physical and mental health of older adults, and the built environment of blocks is the key for cities to cope with aging actively. In addition, mixed land use, pedestrian-oriented design, green space, and aesthetic design may play a role in promoting social interaction. Community design may influence the way people interact within the community and thus affect mental health (12). The characteristics of urban built environments are important predictors of mental health, and many of them have deleterious effects. There is a great need for in-depth research on the different parameters of the built environment and their effects on the mental health of residents at different geographical scales and stages of life. For example, some features of the built environment affect the mental health of residents by exposing them to environmental stresses (e.g., overcrowding, insecurity, etc.) (13). There is consistent evidence that built environment design is associated with promoting active lifestyles that reduce risk factors for chronic disease. In poorer-quality environments, residents were more likely to suffer from depression (14). In addition, the built environment can directly affect the psychological health of residents through their satisfaction with the community environment, Yu et al. (6) found that a number of variables about the living environment, which include safety, maintenance, quiet, housing quality, and greenery, were significantly related to satisfaction; Zhang et al. (15) investigate the relationship between the mental health of low-income residents and their views on their living environment. It is found that good landscape, good house appearance, and good entrance are positively correlated with mental health. On the other hand, the built environment affects the physical and mental health of residents by influencing their travel and physical activities (16). Some studies have proved that the urban form affects residents' travel behavior. Delbosc (17) believed that good transportation conditions improve people's life satisfaction indirectly by improving the accessibility of various resources and, thus, affecting residents' mental health. To sum up, existing studies have confirmed the significant effects of the built environment on residents' mental health, but they mainly focus on urban areas and ignore research on rural areas.

### 2.2. Demographic factors and mental health of the older adult

Studies have proved that demographic factors have a certain impact on the life satisfaction and mental health of older adults (18). A good family atmosphere is very important for older adults. Older married adults have lower depression scores than those who were never married, divorced, or widowed. A good family contributes to the mental health of older adults (19). Social support, such as family members and friends other than spouses, is also important for older adults. Pilar et al. (20) found that social support positively affects the mental health of older adults who do not live with their children. Koh et al. (21) concluded that older adults in Singapore who lived alone and had poor social connections had a higher risk of depression than those who lived with their children. While the influence of living with children on older adults is different, “family support theory” and “family conflict theory” represent two different theoretical orientations of the influence of older adults living style on their mental health (22). According to the former, older adults who live with their children can receive all kinds of support from their children, thus improving their physical and mental health. The latter suggested that conflicts in family life may reduce the benefits of interactions between family members and have a negative impact on older adults (23). Physical health also has a significant impact on the mental health of older adults. Older adults with poor physical health will have more tremendous psychological pressure and mental pressure than those with good physical health, which may cause some mental diseases and affect the mental health of older adults (24). While suffering from chronic diseases will also affect the mental health of older adults, suffering from two or more chronic diseases is positively correlated with the poor psychological health of older adults (25). The mental health status of older adults with chronic diseases is worse than people without disease (26). Some scholars believe that there are higher rates of anxiety and depression in high levels of socioeconomic poverty (27), which increases the risk of depressive events (28) and makes older adults more likely to be admitted to the hospital. These conclusions take into account socioeconomic factors independent of the individual level. Of course, individual or family economic conditions also significantly affect the mental health of older adults. Therefore, this study attempts to explore the effect of the built environment on the mental health of rural older adults after controlling the above demographic variables (29).

## 3. Methodology

### 3.1. Sample selection and data collection

Jintang County is the key development county in the “Chengdu Plain Economic Circle” and the “Characteristic Industrial Development Area” in Chengdu. It is the pilot county of the National Intellectual Property Strength County Project and the typical county of suburban urbanization development in Chengdu. Therefore, this study takes Jintang County as a case study and randomly selects two towns/streets (Guancang and Qingjiang) from 19 towns/streets as sample towns/streets. Through a preliminary survey and a random selection, 11 sample villages were selected.

The formal questionnaire survey was conducted from 5 January 2020 to 10 January 2020. The 10 survey researchers in this research are all postgraduate students from the Chengdu University of Technology. All the researchers had rich experience in village entry and household investigation. In the process of investigation, the 10 researchers were divided into two groups; the researchers scattered around the village committee or senior citizens' activity square and randomly selected villagers over 60 years old for a one-to-one questionnaire survey. The researchers randomly handed out 620 paper questionnaires, 545 were collected, and 30 invalid questionnaires with incomplete information and incorrect filling were excluded. Finally, a total of 515 valid questionnaires were collected, with an effective questionnaire recovery rate of 83.06%. The number of valid samples for each village is shown in Table 1.

**Table 1:** The number of valid samples for each village.

**Village name**	**Number of questionnaire copies**	**Proportion**
Hongqi village	50	9.7%
Quhuang village	45	8.7%
Baimaquan community	51	9.9%
Zhualongxi community	48	9.3%
Shuangyan community	30	5.9%
Ronghua village	42	8.2%
Xinshuinian Shui nian village	45	8.7%
Huapaifang village	50	9.7%
Zhongfu community	55	10.7%
Shuangfeng village	53	10.3%
Qingjiang community	46	8.9%

### 3.2. Variable specification

#### 3.2.1. Demographic variables

The demographic variables used in this study mainly include gender, age, marital status, education level, health status, and chronic disease. Among the respondents, 62.3 percent were women and 37.7 percent were men. In rural areas, most of the older adults over 60 had never been to school (228 people, 44.3%) or only had primary school education (229 people, 44.5%), which is in line with China's social situation before the 1960s. More detailed demographic information of the respondents is shown in Table 2.

**Table 2:** The demographic information of the respondents.

**Demographic variable**	**Variables of types**	**Variable definition**	**Number**	**Percent (%)**	
Gender	Categorical variable	Male	Gender 1	194	37.7
		Female	Gender 2	321	62.3
Age	Sequence variable	60–65	Age 1	121	23.6
		66–70	Age 2	143	27.8
		71–75	Age 3	103	20.0
		76–80	Age 4	70	14.1
		81–90	Age 5	66	12.8
		>90	Age 6	8	1.7
Education	Sequence variable	None	Education background 1	228	44.3
		Primary school	Education background 2	229	44.5
		Junior high school	Education background 3	48	9.2
		Senior high school	Education background 4	8	1.6
		University or above	Education background 5	2	0.4
Marital Status	Categorical variable	Married	Marriage 1	412	80.0
		Spinsterhood	Marriage 2	102	20.0
Physical condition	Sequence variable	Very healthy	Fitness 1	45	8.7
		Healthy	Fitness 2	263	51.1
		Ordinary	Fitness 3	123	23.9
		Bad	Fitness 4	72	14.0
		Very bad	Fitness 5	12	2.3
Chronic disease in oneself	Categorical variable	Have	Chronic disease 1	276	53.7
		Do not Have	Chronic disease 2	239	46.3

#### 3.2.2. Objective built environment variables

The indicators of built environment commonly used in existing studies include density, diversity (30–32), and accessibility (33). This study mainly considers the impact of accessibility to rural older adults' daily activity on their mental health. The researchers used the Ovi Map APP to measure the distances between their homes to their daily destinations by walking, cycling, driving, and public transportation. Overall, rural older adults have the highest walking frequency, and walking promotes physical activity among older adults (34). Therefore, this study took the accessibility index calculation process of walking distance as a reference (35); the calculation formula is as follows:

$$\begin{array}{l} Accessibility\;to\;daily\;destination=\frac{1}{dn\;+\;1} \end{array}$$

In the formula, *d*_*n*_ means the distance from respondents' homes to the nearest daily destination, *n* = 1, 2, 3, 4, 5, 6, 7, 8, 9, 10, 11, 12, 13, and 14. These numbers represent the rural market, health clinic, bus station, main road, activity center for older adults, rural square, supermarket, village committee, kindergarten, primary school, middle school, downtown, town center, and coach terminal.

#### 3.2.3. Subjective perception variable

The perceived built environment has a significant positive effect on the mental health of older adults (36). According to the reference (30), 20 built environment perception items and eight travel preference items were used in this study, and a 5-Likert scale was used to measure respondents' recognition degree of the above items. Exploratory factor analysis was used to analyze the above perception items. Two factors of the 20 built environment items with a load <0.4 were excluded. The results showed that *P* = 0.000 and KMO = 0.837, which means that all the built environment perception items are suitable for factor analysis. Finally, four common factors were extracted from the 18 items, namely, well-constructed roads, safe neighborhoods, convenient transportation, and feeling good to walk. The factor analysis results of the subjective built environment are shown in Table 3.

**Table 3:** Factor analysis result of built environment perception.

**Composition**				
**Factor**	**Well-constructed roads**	**Safe neighborhood**	**Convenient transportation**	**Feeling good to walk**
There are good sidewalks to go around	0.788	0.196	0.190	0.021
There are good bike paths to go around	0.641	0.007	0.097	−0.278
There are good motorways to go around	0.755	0.276	0.213	−0.016
It is convenient to go to the bus station	0605	0.189	0.270	0.10
It is convenient to get to my daily destinations	0.720	0.342	0.247	0.137
Public facilities maintenance service is very good	0.707	0.321	0.271	0.070
Daily travel is safe	0.557	0.625	0.135	0.046
Travel road is broad	0.571	0.630	0.074	0.066
Travel road is smooth	0.591	0.616	0.026	0.097
Daily travel is not congested	0.475	0.630	0.125	0.105
There have been no traffic accidents near my home	0.206	0.808	0.089	0.037
Daily necessities can be bought within a short distance of my home	0.202	0.675	0.212	0.109
There is no crime in my neighborhood	0.028	0.779	0.227	−0.219
Riding a motorcycle is quick and convenient	0.063	−0.050	0.761	0.015
Riding a bike is quick and convenient	0.230	0.206	0.735	−0.071
Riding a tricycle is convenient and easy	0.271	0.258	0.648	0.069
Other travel modes are also quick and convenient	0.357	0.341	0.613	0.134
Walking is good for exercise, and it is cheap and convenient	0.021	0.024	0.059	0.941
Total variance interpretation				
Characteristic value	8.066	1.508	1.246	1.086
Percentage of variance (%)	44.812	8.379	6.920	6.031
Cumulative percentage of Variance (%)	44.812	53.191	60.111	66.143

The result of exploratory factor analysis on travel preference showed that *P* = 0.000 and KMO = 0.805. Two common factors were extracted from the eight items, namely, vehicle preference and walking preference, with a total interpretation rate of 60.959%. The factor analysis results of travel preference are shown in Table 4 below.

**Table 4:** Factor analysis results of travel preference after rotation.

**Component**		
**Factor**	**Vehicle preference**	**Walking preference**
I like driving	0.769	0.209
I like taking public transport	0.653	0.164
I like riding a battery bike	0.726	−0.057
I like riding motorcycles	0.852	0.198
I like riding a bicycle	0.740	0.189
I like riding a tricycle	0.523	0.422
I like walking	−0.046	0.825
I prefer other ways to get around	0.359	0.638
Total variance interpretation		
Characteristic value	3.627	1.009
Percentage of variance (%)	45.340	15.619
Cumulative percentage of variance (%)	45.340	60.959

#### 3.2.4. Mental health variables of rural older adults

In this study, the WHO-5 scale was used to measure the mental health status of rural older adults. It had five items measured by a 5-point Likert scale. The total score is 25 points, and a score ≥13 points indicates that the respondent is mentally healthy. The survey results showed that 69.7% of the respondents were <13 points, indicating that the mental health problems of rural older adults are prominent. Thus, the mental health status of older adults is a binary variable, healthy and unhealthy.

## 4. Results and discussion

### 4.1. Goodness-of-fit test

The Binary Logistic Regression Model was selected to explore the influence of the rural built environment on the mental health of older adults after controlling demographic information. In this study, stepwise regression was adopted. Firstly, demographic variables were added to Model 1, and then subjective built environment, travel preference, and the built environment variables were added to the binary logistic regression step by step. Nagelkerke *R*^2^ and −2LL and Hosmer-Lemeshow were used to check the model's goodness of fit. As the variables continued to be added to the model, Nagelkerke *R*^2^ gradually increased, and the value of −2LL (−2 logarithmic likelihood ratio) gradually decreased, which showed that all variables contributed well to the model. It indicates that the models fit the data well. More details about the goodness of fit for the binary logistic regression are shown in Table 5.

**Table 5:** Goodness of fit of binary logistic regression models.

**Model**	**Nagelkerke *R*^2^**	**−2 LL**	**HL (*P*-value)**
Model 1	0.142	575.882	5.780 (0.672)
Model 2	0.222	542.617	10.372 (0.240)
Model 3	0.328	494.624	17.078 (0.089)
Model 4	0.470	423.158	5.795 (0.670)

In addition, the variance inflation factor (VIF) was used to test multicollinearity. The larger the VIF was the stronger the multicollinearity was, and the maximum critical value of VIF was 10 (37). The VIF values in this study are all < 10. Therefore, there is no multicollinearity problem between variables in this study, and all the VIF values are shown in Table 6.

**Table 6:** Results of binary logistic regression.

**Variable**	**Model 1**	**Model 2**	**Model 3**	**Model 4**	**VIF value**
**Demographic information**					
Gender	0.180	0.169	−0.076	−0.297	1.204
Chronic disease	0.336	0.194	−0.075	0.043	
Marital status	0.865^**^	0.850^**^	0.848^**^	0.693^*^	1.258
Age	0.046	0.032	0.087	0.189^**^	1.393
Education	0.240^*^	0.210	0.236	0.333^*^	1.169
Physical Condition	0.548^***^	0.477^***^	0.642^***^	0.693^***^	1.221
**Built environment perception**					
Well-constructed roads		0.304^**^	0.413^***^	0.289^**^	1.170
Safe neighborhood		0.522^***^	0.669^***^	0.547^***^	1.244
Convenient transportation		0.208	0.138	0.024	1.117
Feeling good to walk		0.172	0.142	0.019	1.096
**Travel preference**					
Vehicle preference			0.778^***^	0.929^***^	1.156
Walking preference			0.337	0.337	1.126
**Objective built environment**					
Rural market accessibility				3.918^**^	5.272
Health clinic accessibility				2.733^*^	2.322
Bus stop accessibility				−2.771^**^	2.022
Main road accessibility				1.107^**^	1.577
Activity center for older adults accessibility				−0.739	3.009
Rural square accessibility				0.311	1.618
Supermarket accessibility				1.411^*^	2.053
Village committee office accessibility				1.859^*^	2.393
Kindergarten accessibility				1.482	2.537
Secondary school accessibility				0.172	5.304
Country center accessibility				1.183	1.901
Town center accessibility				−4.879^***^	2.734
Coach terminal accessibility				−3.827^**^	1.372

N = 515;

*** P < 0.01,

** P < 0.05,

* P < 0.1.

### 4.2. Results of binary logistic regression

SPSS24.0 was used to fit the binary Logistic regression model, and it was found that most of the factors considered in this study had a significant impact on the mental health of rural older adults. The results of binary logistic regression are shown in Table 6.

### 4.3. Effects of demographic variables

Regarding demographic variables of the respondents, physical health, education level, and marital status have significant effects on the mental health of rural older adults. Married rural older adults have a greater probability of mental health than unmarried older adults (divorced, widowed, and unmarried) (B = 0.865; *P* < 0.05). We conclude that unmarried older adults have a higher probability of negative emotions than married older adults, which is consistent with the research results of existing studies (19). There is a significant positive correlation between the mental health of rural older adults and the level of education (B = 0.240, *P* < 0.1), indicating that in terms of mental health, older people with higher education are better than those with lower education (38).

With the increase in age, the physical health status of older adults is relatively low. The better the physical health status the higher the mental health (B = 0.548; *P* < 0.01). This result is similar to the conclusion of previous studies, indicating a significant correlation between residents' physical health and mental health, and older adults with better physical health status have better mental health status (25). To sum up, this study found that age, physical condition, and marital status of older adults have significant effects on the mental health of older adults.

### 4.4. Effects of subjective perception variables

Built environment perception and travel preference are subjective perceived factors. Studies have shown that safe, accessible, comfortable, and beautiful streets and public spaces are important places for residents' daily life and neighborhood communication (39). After controlling demographic variables, this research found that when rural older adults perceive well-constructed road (B = 0.304, *P* < 0.05), they were more likely to be mentally healthy. Li et al. (40) believe that older adults living in communities with well-constructed roads have higher levels of social activities, which is conducive to improving their mental health levels. In addition, better security in rural areas was beneficial to the mental health of rural older adults (B =0.522; *P* < 0.01). Residential security is one of the factors affecting residents' mental health. High crime rates nearby will reduce residents' sense of security and cause certain mental burdens (15).

As for travel preference, this study found that the more older adults prefer to travel using transportation, the better their mental health level (B = 0.778, *P* < 0.01). Related studies have also found that if older adults can travel by vehicle, their frequency of outdoor travel will be increased, and the purpose of encouraging older adults to participate actively in society will be achieved, which will promote their happy mood and benefit their mental health (41).

### 4.5. Effects of objective built environment

Among the accessibility indicators of 13 destinations of rural residents' daily activities, eight indicators significantly impact the mental health of rural older adults. Specifically, accessibility to the rural market (B = 3.918, *P* < 0.05), the health clinic (B = 2.733, *P* < 0.1), the bus station (B = 2.771, *P* < 0.05), the village committee office (B = 1.859, *P* < 0.1), the supermarket (B = 1.411, *P* < 0.1), and the main road (B = 1.107, *P* < 0.05) were positively correlated with the mental health of rural older adults. While accessibility to the town center (B = −4.379, *P* < 0.01) and the coach terminal (B = −3.827, *P* < 0.05) had a significant negative impact. It is proved that the better the accessibility to daily activity destinations, the more beneficial the mental health of rural older adults (42). Among them, accessibility to the market, health clinic, and public transport station play a prominent role because the market is the central place for villagers' daily trade, which is necessary for daily activity and has become a regular activity of the villagers (42). With the increase in age, older adults in rural areas have a higher demand for health services (42). At present, rural health clinics are the most important daily health service stations for rural residents. Therefore, the convenience and accessibility of rural health stations are essential for rural older adults, which can effectively reduce the psychological pressure on rural older adults in seeking medical treatment. Easy accessibility of public transport stations contribute to older adults' travel satisfaction and use frequency (6), which is also conducive to rural older adults' participation in social activities and their mental health (23). In addition, accessibility of the village committee office, supermarkets, and main roads also significantly positively impacted the mental health of rural older adults. The accessibility of the town center and coach terminal beyond the scope of the township and village significantly negatively impacts the mental health of rural older adults. According to the random interview, the rural town center is an important place for daily life which is a commercial place. The coach terminal and the town center both have the characteristics of loud environmental noise, which affects the daily life of nearby residents and is not conducive to their mental health.

## 5. Conclusion

The problem of the aging population is becoming more and more serious. This study focuses on the mental health of rural older adults and attempts to explore the effects of a rural built environment on the mental health of rural older adults after controlling demographic variables. Through one-to-one and face-to-face questionnaire surveys in rural areas, 515 valid samples were obtained. Factor analysis and binary logistics regression were used in this study. The results showed that: (1) Gender, education level, marital status, chronic disease, and physical health status of rural older adults significantly affected their mental health. (2) Rural older adults perceived that good road conditions and safe living environments had positive effects on their mental health, and older adults who prefer to travel by vehicle have better mental health. (3) Most of the accessibility indicators significantly positively affect the mental health of rural older adults except for town center and coach terminal accessibility.

Based on the above research results combined with the current situation of rural older adults in Sichuan, this paper proposes the following suggestions for improving the mental health level of rural older adults. (1) Relevant government departments should pay more attention to rural older adults who are divorced, living alone, and other empty nesters. (2) Further improvement of rural road traffic and living environment should be given constant attention. (3) Daily activities destination of villagers should be further optimized according to the needs of older adults to create a rural living environment suitable for aging.

## Data availability statement

The raw data supporting the conclusions of this article will be made available by the authors, without undue reservation.

## Ethics statement

Ethical review and approval were not required for the study on human participants in accordance with the local legislation and institutional requirements. The patients/participants provided their written informed consent to participate in this study.

## Author contributions

Conceptualization and draft writing: PL and YW. Resource: TW. Data analysis: PL, YW, and TW. All authors contributed to the article and approved the submitted version.
